# Assessment of the dietary and nutritional status of Iranian pregnant women: a study employing both a priori and a posteriori analytical approaches

**DOI:** 10.1038/s41598-026-51749-8

**Published:** 2026-05-21

**Authors:** Zahra Kashi, Akbar Fazeltabar Malekshah, Mina khasayesi, Mohammad Heidari Seyedmahalleh

**Affiliations:** 1 Diabetes Research Center, Imam Teaching Hospital, Mazandaran University of Medical Sciences, Sari, Iran; 2 Diabetes Research Center, Institute of Herbal Medicines and Metabolic Disorders, Sari Imam Khomeini Hospital, Mazandaran University of Medical Sciences, Sari, Iran

**Keywords:** Dietary patterns, Pregnant women, Gestational, Nutrient adequacy, Iran, Mediterranean Diet Score, Principal component analysis, Na/K ratio, Diet quality, Nutrient-rich food index, Mean adequacy ratio, Total antioxidant capacity, Diseases, Health care, Medical research, Risk factors

## Abstract

Dietary pattern analysis provides a holistic assessment of diet quality critical for maternal-fetal health, offering insights superior to single-nutrient evaluation. Hereby, we aimed to concurrently evaluate established a priori and data-driven a posteriori dietary patterns, alongside nutrient-mineral adequacy, among pregnant women. This cross-sectional study recruited 233 women in their first trimester from “Mazandaran, Iran” province between 2020 and 2023. Dietary intake was measured via a validated 168-item Food Frequency Questionnaire. Adherence to predefined guidelines was assessed using a modified 8-point Mediterranean Diet Score (MDS) and three Nutrient Rich Food (NRF) indices (NRF6.3, NRF9.3, NRF15.3). Empirical patterns were derived using Principal Component Analysis (PCA) on 26 food groups via R Studio. Nutritional status was quantified using the Mean Adequacy Ratio (MAR), Protein Adequacy Ratio (PAR), and adequacy metrics for other nutrients/minerals. The study exhibited high metabolic risk factors in pregnant women, with 61.0% overweight/obese and 59.2% vitamin D deficient. Overall diet quality was suboptimal, evidenced by low MDS (Mean = 3.35) and poor micronutrient density (Mean MAR = 0.624), signifying that diets met only approximately 62% of recommended intakes. PCA identified a healthy “Prudent” pattern (PA2), a mixed “Traditional” pattern (PA1), and an unhealthy “Western” pattern (PA3). Increased adherence to the Prudent pattern significantly correlated with improved MAR, PAR, and a desirable decrease in Na/K ratio. Conversely, adherence to the Beverage & Fast-food pattern was associated with pervasive declines in all adequacy markers, including a severely decreased MDS (β = -0.26, p-value = < 0.001). Pregnant women in this regional study exhibit critically suboptimal dietary quality and significant micronutrient shortfalls, largely driven by low adherence to prudent, nutrient-dense patterns, and the majority depend on unhealthy food choices.

**Supplementary Information:**

The online version contains supplementary material available at 10.1038/s41598-026-51749-8.

## Introduction

Diet is increasingly evaluated as a whole-of-diet exposure rather than as a set of isolated nutrients^1^. Studies show that involving more aspects of dietary habits and choices has made progress in understanding both nutritional and dietary modifications needed to prevent diseases^2^. People eat foods in combinations, and nutrients interact biologically; thus, dietary pattern analysis can capture the cumulative and potentially synergistic effects of the habitual diet on health better than single-nutrient or single-food approaches^3^.

Since fetal growth is largely dependent on the nutrients provided by the mother, inadequate maternal diet and unhealthy lifestyle habits can adversely influence pregnancy outcomes and the long-term health of the child^4^. Healthy dietary patterns—characterized by higher intakes of vegetables, fruits, legumes, whole grains, fish, and plant oils—are consistently associated with better health profiles in reproductive-age women and during pregnancy^5^. Observational syntheses show that such patterns are linked to lower odds of hypertensive disorders of pregnancy (HDP), gestational diabetes mellitus (GDM), and preterm birth (PTB), and support healthy fetal growth trajectories into infancy and beyond^6^.

Two methodological families dominate dietary pattern research. A priori (hypothesis-driven) indices score adherence to pre-set recommendations or cultural models whereas A posteriori (data-driven) methods, such as principal components analysis (PCA), factor analysis, or clustering, extract patterns from observed intake correlations in a given population^7^. Each has strengths and limitations: a priori indices are comparable across studies but may fail to capture local eating cultures; posteriori methods are population-specific and can reveal novel, contextually meaningful patterns but are not standardized^1,7^. Applying both within the same dataset can provide complementary insight into diet quality and the empirical structures that underlie it^8^.

Despite the overall benefits of “healthy” patterns, specific findings by population, trimester, and outcome remain heterogeneous^6^. Meta-analyses report protective associations for healthy patterns and higher risks with “Western” or processed patterns, yet some cohorts (or specific indices) show null or context-dependent effects^9^. Critically, much of the evidence emphasizes a priori indices in developed western cultures and hence the empirical findings remains relatively sparse especially in developing and traditional societies^9^. This gap is particularly salient for understanding diet quality among pregnant women in settings where cultural foodways and food environments differ from Western norms. By analyzing both A priori and A posteriori dietary patterns in the same cohort of pregnant women—and placing these results alongside the broader literature—this study contributes to that underexplored space.

## Methods

### Study design and participants

This cross-sectional study was carried out from March 2020 to February 2023 in “Mazandaran, Iran” province, northern Iran. Volunteers were recruited from public prenatal clinics affiliated with Mazandaran University of Medical Sciences, which are primarily located in the city of Sari. After steps of exclusion, the ultimate number of 233 pregnant women enrolled in the study, as presented in the participant flow chart (Supplementary Chart 1). Participants were all in their first trimester, and the study employed systematic random sampling. Each qualified pregnant woman was assigned to a number and through random number selection, the enrolled participants were chosen. Being under 14 weeks pregnant, having permanent residency in “Mazandaran, Iran” for at least six months, and being willing to provide informed consent were our main inclusion necessities. Exclusion criteria included a medication for chronic diseases (renal, hepatic, or thyroid disorders), conditions affecting dietary intakes (having difficulties eating or being on appetite-lowering medications), being consulted for any sort of diets or special diets (Low Carbohydrate, Fasting, Vegan, and any kinds of Calorie Deficit diets). Patients whose dietary energy report was either overreported or underreported (800 kcal/day to 5000 kcal/day) were not included in the study^10^. This study was approved by the Ethics Committee of Mazandaran University of Medical Sciences (Approval ID: IR.MAZUMS.REC.13984629). Written informed consent was obtained from all participants before enrollment. Participants identified with severe vitamin deficiencies (Energy, Macro/Micro nutrients, and minerals) were provided counseling and referred to a clinical nutritionist for further evaluation and appropriate supplementation, following ethical clinical care protocols. Confidentiality and anonymity of participant data were strictly maintained throughout the study.

### Assessment and measurements

Information on maternal age, education, employment status, and household socioeconomic background was collected using a structured questionnaire. This tool had previously been validated and applied in the Iranian Surveillance of Risk Factors of Non-communicable Diseases (ISRFNCD) study^11^. The questionnaire covered variables such as age, sex, education, household conditions, marital and occupational status, disease duration, treatment method, and comorbidities. Family Economic status was basically calculated through the summed income of both participant and her husband and scaled by a Z score approach. The participants below the 0 were defined as “Low Income”, participants with Z score between 0 and 1 as “Medium Income”, and participants above 1 were considered “High Income”^11^. Anthropometric data, including pre-pregnancy body weight and height, were measured to calculate body mass index (BMI) as weight in kilograms divided by height squared in meters (kg/m²). Mothers’ pre-pregnancy weight was either self-reported or extracted from pre-pregnancy medical history and anthropometric assessments. Women were categorized as underweight (< 18.5 kg/m²), normal weight (18.5–24.9 kg/m²), overweight (25–29.9 kg/m²), or obese (≥ 30 kg/m²). Metabolic Syndrome (MetS) components were adapted from a Joint Interim Statement published in 2009, in a way that any of the components met the criteria^12^.

For biochemical analysis, a 12-hours fasting venous blood sample (5 mL) was collected from each participant. Serum concentrations of 25-hydroxyvitamin D [25(OH)D], Zinc, calcium, and phosphorus were determined using a chemiluminescent immunoassay (CLIA), a method with proven reliability. Measurements were carried out using high-sensitivity commercial kits (DiaSorin, Italy) on the LIAISON XL analyzer. Samples were centrifuged, and the separated serum was stored at − 80 °C until testing. The assay had a coefficient of variation (CV) below 8%, and the lower limit of detection for 25(OH)D was 4 ng/mL, using a calibrated Shimadzu AA-670 spectrophotometer with a detection limit of 1 µg/dL and a coefficient of variation < 5% for Zinc concentration, ensuring both accuracy and reproducibility. All analyses were performed at the Central Clinical Laboratory of Imam Khomeini Teaching Hospital in Sari (Mazandaran, Iran). Vitamin D status was categorized according to Institute of Medicine (IOM) guidelines and related literature^25^ as follows: severe deficiency (< 10 ng/mL), deficiency (10–20 ng/mL), insufficiency (21–29 ng/mL), and sufficiency (≥ 30 ng/mL)^13^. Zinc status was classified according to cut-off values proposed by the International Zinc Nutrition Consultative Group (IZiNCG) and WHO guidelines for pregnant women: Zinc Deficiency (SZnD): < 10 ng/mL ; Zinc Sufficiency (SZnS): ≥10 ng/mL^14^.

### Dietary assessment and food grouping

Dietary intake was evaluated using a semi-quantitative food frequency questionnaire (FFQ) that included 168 items and had been previously validated for use in the Iranian population^15,16^. Nutritional information was obtained through face-to-face interviews conducted by a trained nutritionist. Participants were asked to report the frequency and portion size of each food item consumed over the preceding 12 months, thereby capturing their habitual dietary patterns across different seasons of the year. Once the raw dietary data were collected, daily intakes of individual food items were computed. Reported portion sizes were converted into gram equivalents using a standardized reference manual of household measures^17^, which provides culturally adapted examples for common Iranian foods and beverages. The nutrient composition and caloric values of the reported foods were primarily determined using the United States Department of Agriculture (USDA) food composition database, as incorporated in the Nutritionist IV software (First Databank, Hearst, San Bruno, CA, USA). To address limitations in the USDA database regarding traditional Iranian foods, the Iranian Food Composition Table^18^ was also consulted. This dual approach allowed for a more precise estimation of macronutrient and micronutrient intake, particularly for regional dishes and food products that are not represented in international databases. In addition to estimating total energy intake, the nutrient analysis included key macronutrients (carbohydrates, proteins, fats) and micronutrients of relevance to maternal and fetal health, such as calcium, iron, zinc, folate, and vitamins.

### A priori dietary patterns and nutritional adequacy indices

We calculated several indices to assess the adequacy of nutrient intake in these pregnant women, focusing on both overall micronutrient sufficiency and key nutrient balance ratios. All adequacy metrics were based on recommended dietary allowances (RDA) or analogous standards for pregnant women and were computed using each participant’s daily intake as estimated by the Nutritionist IV analysis. Mediterranean Diet score (MDS), Nutrient-Rich Foods Index (NRFI) along with nutritional adequacy indices like Mean Adequacy Ratio (MAR), Protein Adequacy Ratio (PAR), Dietary TAC (Total Antioxidant Capacity), MUFA/SFA ratio (Monounsaturated Fat to Saturated Fat), Ca/P ratio (Calcium-to-Phosphorus), Na/K ratio (Sodium to Potassium), B9/B12 ratio (Folate to Vitamin B12) were applied as A priori indices^19,20^. Definition and calculation methods are presented in supplementary materials (ST1-3).

### A posteriori pattern derivation

To derive dietary patterns empirically (a posteriori), we performed principal component analysis (PCA) on the FFQ intake data. To define components required for PCA, we first aggregated the FFQ items into twenty-six food groups (based on nutritional similarity or culinary usage) and then used the intake of each food group as the variable. Dietary patterns were extracted by examining the correlation structure of intakes and identifying principal components with eigenvalues > 1.0 (Kaiser’s criterion)^21^. We retained all components with eigenvalues greater than one for interpretation, and we also evaluated the scree plot and factor interpretability to determine the final number of patterns to retain. The retained components were then orthogonally rotated using Varimax rotation to achieve a simpler structure with uncorrelated factors. After rotation, each pattern was characterized by a set of foods or food groups with high factor loadings (Loading value ≥ 0.3). Hyperparameter tuning for the PCA development is presented in (ST4.).

### Data preprocessing, variable computation, and statistical modeling software

We applied the latest versions of Excel (Microsoft Corporation. Microsoft Excel [Internet]. 2018. Available from: https://office.microsoft.com/excel) and SPSS (IBM Corp. (2020). IBM SPSS Statistics for Windows (Version 27.0) [Computer software]. IBM Corp.), for data preparation and preprocessing, including:^1^ importing data from questionnaires and data collection staff;^2^ cleaning the dataset from missing, outlier, and mismatched data;^3^ generating new variables via variable computation and classification;^4^ calculating basic statistical reports like means and standard deviations and percentages. Moreover, for data-driven pattern extraction, correlational heatmap figure generation, and Ordinary Least Squares (OLS) linear regression a R Studio (Posit team (2025). RStudio: Integrated Development Environment for R.) was exploited.

## Results

### General characteristics

We analyzed cross-sectional dietary data in 233 pregnant women. The mean age of the participants was 29.5 years. Pre-pregnancy BMI classification indicated that over one-third of the women were overweight (34.8%) and about one-quarter were obese (26.2%), whereas only 2.6% were underweight. Micronutrient deficiencies were notable: although 91.8% had sufficient serum zinc, a majority (59.2%) were vitamin D deficient. Most women (87.1%) had normal fasting blood glucose, but 12.9% showed elevated levels. Almost half of the participants (53.6%) reported using nutritional supplements. The study population was predominantly of lower educational attainment (76% did not have a university degree), and most were not formally employed (85.8%). Socioeconomically, 60.9% of the women were classified as low income. In terms of metabolic health, 58% had no components of metabolic syndrome, 31% had one component, and only ~ 3% of participants had three or more components diagnosed before the pregnancy (Table 1).

**Table 1:** General characteristics of 233 pregnant women enrolled in the study.

Variables ^a^		Frequency ^b^
Age (years)		29.5 6 (2.5)
BMI (kg/m^2)^	UW	
	NW	85 (36.5)
	OW	81 (34.8)
	Obese	61 (26.2)
SZn (ng/mL)	Deficient	19 (8.2)
	Sufficient	214 (91.8)
VD (ng/mL)	Deficient	138 (59.2)
	Sufficient	95 (40.8)
FBS (mg/dL)	Normal	203 (87.1)
	High	30 (12.9)
Supplementation	Yes	125 (53.6)
	No	108 (46.4)
Education	Under Academia	177 (76)
	Bachelor	31 (13.3)
	Master	25 (10.7)
Occupation	Not employed	200 (85.8)
	Employed	33 (14.2)
Family economic status	Low	142 (60.9)
	Medium	75 (32.2)
	High	16 (6.9)
History of MetS	None	135 (57.9)
	One component	73 (31.3)
	Two components	17 (7.3)
	Three components	6 (2.6)
	Five components	2 (0.9)

Body mass index (BMI), VD (Vitamin D), serum zinc (SZn), fasting blood sugar (FBS).

^a^All variables are referred to at the time of data collection, except for BMI, which is based on prepregnancy assessment.

^b^Data presented as: Number (percent of total).

### Dietary profile: food groups

Refined grains were the principal staple in these pregnant women’s diets, with an average intake of ~ 26% of daily calories. Fruits contributed substantially to the diet (~ 11% of energy), alongside dairy products – approximately each contributing ~ 11–12% of caloric intake. Nuts and seeds were also a notable source of energy (7.2% of calories). In contrast, vegetable intake accounted for only ~ 0% of daily calories due to low energy density. Several nutrient-dense or protein-rich foods were consumed in very low quantities: whole grains and legumes each provided < 1% of energy, and fish intake was negligible (0.5% of calories). Consumption of red meat and poultry was modest (1–2% of energy). The participants obtained around 3.7% of their daily calories from added sugars and ~ 5.3% from sweets and desserts, on average. Intake of sugar-sweetened beverages was relatively low (3.3% of energy), and while nearly 1 glass of tea/coffee was consumed daily, this contributed essentially no calories (Table 2). Overall, the dietary profile is characterized by high refined carbohydrate and dairy intakes, with low consumption of whole grains, legumes, and fish.

**Table 2:** Daily consumption of each food group in relation to total calorie intake.

Food group	TEI fraction^a^ (%)	Food group	TEI fraction^a^ (%)
Fruits	11	Fish	0.5
Vegetables	0.1	Eggs	1.3
Whole grains	0.5	Oils (olive/veg)	1.7
Refined grains	26	Animal fat	1.2
Potatoes	3.4	Butter/processed fat	0.3
Legumes	0.5	Added Sugars	3.7
Nuts & seeds	7.2	Sweets & desserts	5.3
Low-fat dairy	11.5	Juice	1
High-fat dairy	12	SSB	3.3
Red meat	2	Tea/coffee	0
Processed meat	1	Salty snacks	4
Fast foods	0.6	Pickles/condiments	0.1
Poultry	1.5	Added salt	0

Total energy intake = TEI; sugar-sweetened beverages = SSB.

^a^ Calorie percent of daily calorie intake.

### Intra-correlations between food groups

**Fig. 1:**
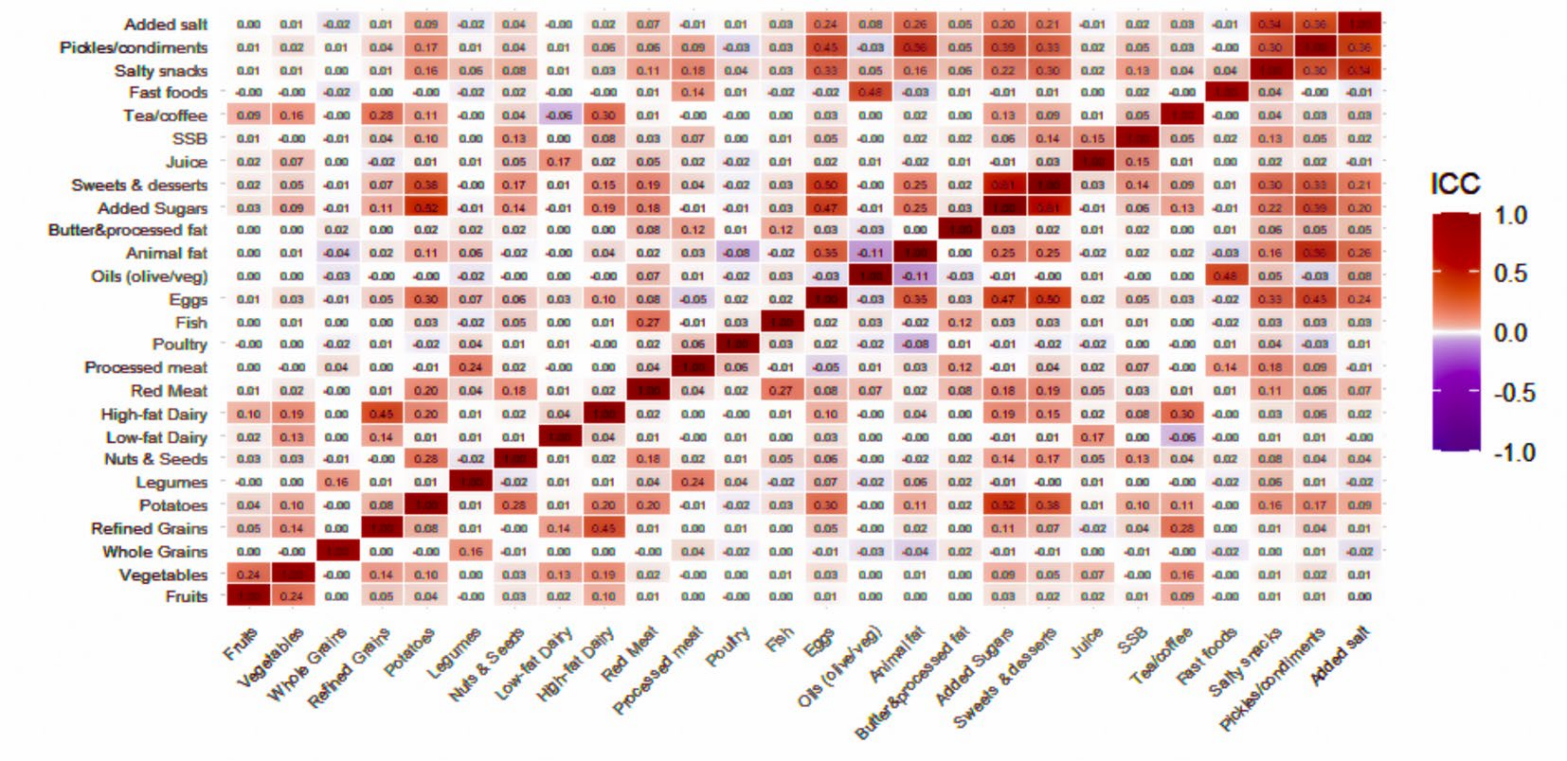
Heatmap of intra-correlation coefficients (ICCs) between food groups.

To assess the correlation between the g/day consumption of each food group, an Intra-Correlation Analysis (ICC) was performed (Fig. 1). The closer the correlation coefficient, the more correlated they are, and the positive or negative sign shows the direct or indirect relationship. Given the absolute value of the coefficient, the intensity of the correlation between food groups inside each pattern is definable which a PCA pattern itself is incapable of revealing it. As demonstrated in ICC analysis, fruits and vegetables were merely correlated with each other and did not present any significant correlations with other food groups (ICC = 0.24). Refined grains were consumed moderately in company with High-fat dairies (ICC = 0.45). Potatoes were significantly associated with added sugar (ICC = 0.52) and moderately with sweets & deserts (ICC = 0.38). On the other hand, these two were significantly linked to more daily consumption of eggs (ICC: sweets & deserts = 0.50; added sugar = 0.47). Regarding the sodium-rich sources, salty snacks were correlated with pickles/condiments (ICC = 0.3) and significantly added salt (ICC = 0.54). Added sugar was also mostly accompanied by more sweets & dessert consumption (ICC = 0.61).

### Nutritional adequacy metrics

Several key nutrient density ratios and adequacy indices were evaluated (Table 3). On average, the women’s diets had a MUFA/SFA ratio slightly above 1 (mean ~ 1.11), with 55.8% of participants achieving a ratio > 1 – indicating monounsaturated fat intake modestly exceeded saturated fat intake in over half of the women. However, the PUFA/SFA ratio was < 1 for 75% of participants (mean ~ 0.81), reflecting a generally low intake of polyunsaturated fats relative to saturates. The Ca/P ratio (calcium-to-phosphorus) averaged ~ 0.93, and 59.2% of women had Ca/*P* < 1, suggesting that calcium intake was often lower than phosphorus intake. The participants’ mean total antioxidant capacity (TAC) z-score was approximately 0.0 (SD ~ 0.66), with about 61% of individuals having a negative TAC z-score (below the reference mean). Folate-to-B12 (B9/B12) ratio had a median value of around 0.006; about 72.5% of participants were above this threshold, implying that the majority had a relatively higher folate intake compared to vitamin B12. Mean adequacy ratio (MAR) was 0.624 ± 0.097, indicating that, on average, dietary intakes met only ~ 62% of the recommended levels across nutrients. Just over half of the women (53.4%) had MAR values below 0.65, signifying suboptimal micronutrient adequacy in more than half the total population. Protein adequacy ratio (PAR) was satisfactory for most: 57.1% had PAR > 1, and the mean PAR was ~ 1.24, although 42.9% did not meet the protein requirement (PAR < 1). The Na/K ratio (sodium-to-potassium) was high, with a mean of ~ 1.56; fully two-thirds of participants (67.4%) had Na/K > 1, far exceeding the ideal of ≤ 1.

**Table 3:** Nutritional assessment of participants based on selected nutritional adequacy metrics.

Nutritional index		Mean (SD)	Cases (%)
TAC Z-score	< 0	-0.463 (0.285)	141 (60.5)
	> 0	0.757 (0.801)	92 (39.5)
	Total	0.019 (0.661)	233 (100)
B9/B12	< 0.006	0.004 (0.0005)	64 (27.5)
	> 0.006	0.007 (0.002)	169 (72.5)
	Total	0.006 (0.002)	233 (100)
Ca/P	< 1	0.781 (0.155)	138 (59.2)
	> 1	1.157 (0.150)	95 (40.8)
	Total	0.934 (0.240)	233 (100)
PUFA/SFA	< 1	0.596 (0.219)	175 (75.1)
	> 1	1.453 (0.464)	58 (24.9)
	Total	0.809 (0.476)	233 (100)
MUFA/SFA	< 1	820 (0.109)	103 (44.2)
	> 1	1.348 (0.344)	130 (55.8)
	Total	1.114 (0.374)	233 (100)
Na/K	< 1	0.485 (0.265)	76 (32.6)
	> 1	2.089 (1.219)	157 (67.4)
	Total	1.565 (1.261)	233 (100)
PAR	< 1	0.769 (0.163)	100 (42.9)
	> 1	1.596 (0.653)	133 (57.1)
	Total	1.241 (0.650)	233 (100)
MAR	< 0.65	0.549 (0.064)	124 (53.4)
	> 0.65	0.710 (0.042)	108 (46.6)
	Total	0.624 (0.097)	233 (100)

Total antioxidant capacity = TAC; Cclcium = Ca; phosphorus = P; polly unsaturated fatty acids = PUFA; mono unsaturated fatty acids = MUFA; sodium = Na, potassium = K; protein adequacy ratio = PAR; mean nutrient adequacy ratio = MAR; standard deviation = SD.

### A priori dietary pattern scores

Four predefined diet quality scores were assessed (Table 4). The Mediterranean Diet Score (MDS) (0–8 scale) was low on average: the mean MDS was 3.35 (SD 1.22), and about half of the women (52.9%) scored < 4, indicating poor adherence to a Mediterranean-style dietary pattern. Only 47.1% achieved an MDS above 4 (Table 4). The Nutrient Rich Food index scores varied by the nutrient components included. NRF6.3 was notably low: the average NRF6.3 score was − 12.61 (SD = 24.3), and a vast majority (87%) of participants had NRF6.3 values below the threshold of 3 (Table 5). In contrast, scores on the broader indices NRF9.3 and NRF15.3 were higher. The mean NRF9.3 was 23.3 (SD = 25.3); 87.4% of women exceeded the modest cut-off of 6. Similarly, NRF15.3 averaged 28.7 (SD = 26.0), with 81.2% of participants scoring above 12.

**Table 4:** A priori dietary patterns scores.

Dietary index		Mean (SD)	Cases^a^ (%)
NRF6.3	< 3	-15.04 (25.15)	194 (87)
	> 3	3.60 (0.5)	29 (13)
	Total	-12.61 (24.28)	223 (100)
NRF9.3	< 6	4.77 (1.01)	28 (12.6)
	> 6	25.97 (25.97)	195 (87.4)
	Total	23.30 (25.29)	223 (100)
NRF15.3	< 12	9.70 (1.61)	42 (18.8)
	> 12	33.07 (27)	181 (81.2)
	Total	28.67 (26)	223 (100)
MDS	< 4	2.40 (0.75)	118 (52.9)
	> 4	4.423 (0.62)	105 (47.1)
	Total	3.35 (1.22)	223 (100)

Nutrient rich foods = NRF; Mediterranean Diet Score = MDS; standard deviation = SD.

^a^ Due to energy adjustment and regarding the over and under estimations, 10 of the participants were ruled out from the dietary pattern analysis (*n* = 223).

**Table 5:** A posteriori dietary patterns and their principal components’ loading values derived from 26 food groups.

Food group	A posteriori diet patterns		
	PA1^a^	PA2^a^	PA3^a^
	Traditional/mixed	Healthy/prudent	Unhealthy/western
Fruits	0.43	0.45	
Vegetables	0.34	0.47	
Whole grains			
Refined grains	0.57		
Potatoes	0.51	0.34	
Legumes			
Nuts & seeds		0.43	
Low-fat dairy			
High-fat dairy	0.69		
Red meat		0.7	
Processed meat			
Poultry			
Fish		0.58	
Eggs	0.77		
Oils (olive/veg)			0.32
Animal fat	0.74		
Butter & processed fat		0.44	
Added Sugars	0.8		
Sweets & desserts	0.67		
Juice		0.31	
SSB			
Tea/coffee	0.43		0.52
Fast foods			
Salty snacks	0.33		0.61
Pickles/condiments	0.81		
Added salt	0.48		0.68
Eigenvalue^b^ (s2)	5.06	2.13	1.6
Proportion of variance^c^ (%)	19.45	8.21	6.16

^a^ Loading value ≥ 0.3 was considered significant.

^b^ Eigenvalue (SUM S2) is the sum of squared loading values inside each PCA pattern.

^c^ Proportion of Variance (%) is one pattern’s coverage of the daily consumption.

### A posteriori patterns: food groups-based

**Fig. 2:**
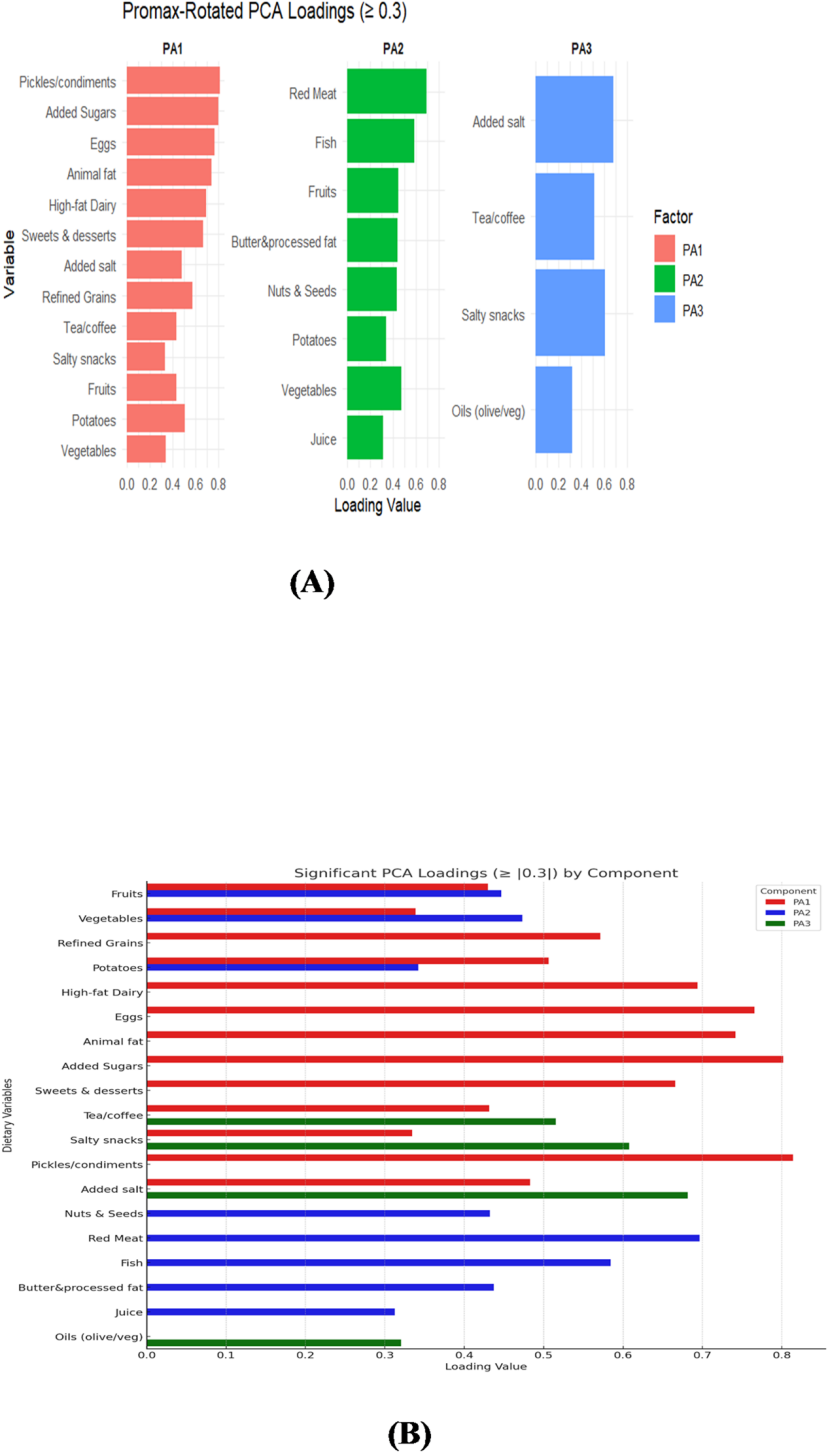
(**A**) Loading values of 26 food groups across the PCA-obtained food patterns (PA1-3). (**B**) A posteriori food patterns via principal component analysis using food groups as components.

In addition to pre-defined food patterns, PCA was conducted using food groups as components, yielding three major patterns: PA1, PA2, and PC2 (Table 5; Fig. 2). These three principal components collectively captured a substantial portion of the dietary variation: PA1 ~ 19.5%, PA2 ~ 8.2%, and PA3 ~ 6.2%. PA1 emerged as an overarching pattern associated with a wide array of food groups: Fruits, Vegetables, Refined Grains, Potatoes, High-fat Dairy, Eggs, Animal fat, added sugar, Sweets & deserts, Pickles/Condiments, and Added salt, attributing to “Traditional” or mixed dietary pattern. A healthier pattern was also obtained. PA2 or conventionally noticed as “Prudent” diet pattern, revealed to be comprised of: Fruits, Vegetables, Potatoes, Nuts & Seeds, Red meat, Fish, Butter, and juices. Although the third pattern occupied the least of the population’s eating habits, it was associated with some unhealthy components, making it the most fitted pattern for “Western”. Principal components are: Tea/Coffee, Salty snacks, Added salt, and Oils (Table 5; Fig. 2).

### Internal validity: nutritional trends across pattern tertiles

**Fig. 3:**
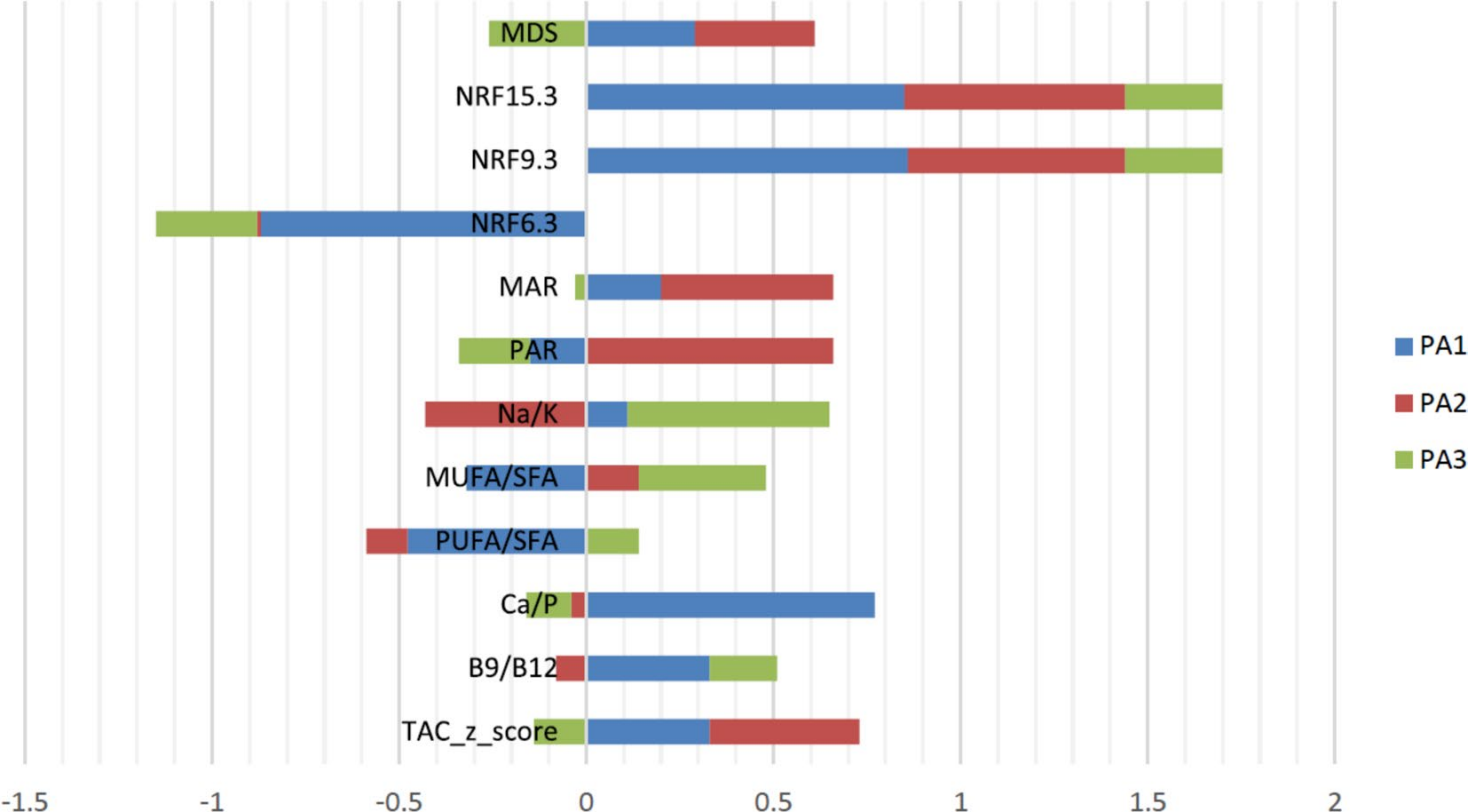
Trends of nutritional status reflecting indices and a priori dietary patterns among tertiles of PCA-driven dietary patterns.

We evaluated how various nutrient indices and diet quality scores changed across increasing tertiles of each PCA-derived pattern. This analysis revealed that certain patterns were associated with markedly improved nutritional profiles from the lowest to the highest tertile, while others corresponded to deteriorations in diet quality (Table 6). For instance, PA1 (Traditional pattern) showed a mixed trend across tertiles. women in the highest PA2 tertile had significantly lower PUFA/SAF (β = -0.48, p-value = < 0.001), MUFA/SFA (β = -0.32, p-value = 0.016), and PAR (β = -0.15, p-value = 0.032), however significant superiorities for B9/B12 ratio (β = 0.33, p-value = < 0.001), MAR (β = 0.2, p-value = 0.021), and MDS (β = 0.29, p-value = 0.034). Also, the “Prudent” pattern demonstrated uniformly favorable shifts: moving from the first to third tertile of PA2, participants showed progressive increases in TAC, MAR, PAR, NRF9.3, and NRF15.3 ratio, alongside a decrease in Na/K (β = -0.43, p-value = < 0.001). In addition to all, the most Mediterranean diet-friendly pattern was declared to be PA2 (β = 0.32, p-value = 0.008). In contrast, PA3 or “Western” pattern tertiles were associated with both an increase in Na/K ratio (β = 0.54, p-value = < 0.001) and a decrease in PAR (β = -0.19, p-value = < 0.001), TAC (β = -0.14, p-value = 0.004), and MDS (β = -0.26, p-value = < 0.001). Surprisingly, elevation in NRF9.3 and NRF15.3 scores, along with a decrease in NRF6.3, was significant across the tertiles of all three diet patterns (Fig. 3).

**Table 6:** Trends of predefined food patterns and nutritional indices from the first towards the third tertile of PCA-driven patterns defined by Linear Regression correlation.

PCA pattern		TAC	B9/B12	Ca/*P*	PUFA/SFA	MUFA/SFA	Na/K	PAR	MAR	NRF6.3	NRF9.3	NRF15.3	MDS
PA1	β-coefficient^a^	0.33	0.33	0.77	-0.48	-0.32	0.11	-0.15	0.2	-0.87	0.86	0.85	0.29
	p-value^b^	**< 0.001**	**0.012**	0.561	**< 0.001**	**0.016**	0.389	**0.032**	**0.021**	**< 0.001**	**< 0.001**	**< 0.001**	**0.034**
PA2	β-coefficient^a^	0.4	-0.08	-0.04	-0.11	0.14	-0.43	0.66	0.46	-0.01	0.58	0.59	0.32
	p-value^b^	**< 0.001**	0.494	0.712	0.357	0.255	**< 0.001**	**< 0.001**	**< 0.001**	0.896	**< 0.001**	**< 0.001**	**0.008**
PA3	β-coefficient^a^	-0.14	0.18	-0.12	0.14	0.34	0.54	-0.19	-0.03	-0.27	0.26	0.26	-0.26
	p-value^b^	**0.004**	**0.019**	0.061	0.078	**< 0.001**	**< 0.001**	**< 0.001**	0.531	**< 0.001**	**< 0.001**	**< 0.001**	**< 0.001**

Principal Component Analysis = PCA; Total Antioxidant Capacity = TAC; Calcium = Ca; Phosphorus = P; Polyunsaturated Fatty Acids = PUFA; Mono Unsaturated Fatty Acids = MUFA; Sodium = Na, Potassium = K; Mean nutrient Adequacy Ratio = MAR; Protein Adequacy Ratio = PAR; Standard Deviation = SD.

^a^ β-coefficient is – or + if the correlation is inverse or direct; and is more empowered if the absolute value increases.

^b^ p-value obtained from ordinary least squares (OLS) linear regression, treating tertile as a numeric predictor and daily calorie intake as a confounder (Energy-adjusted) to assess the linear trend in the continuous outcome.

## Discussion

This study aimed to examine dietary patterns and nutritional adequacy among pregnant women in northern Iran using a dual approach. Specifically, we combined a priori diet quality indices—including the MDS and NRF indices—with a posteriori patterns derived through PCA. By utilizing these complementary methods, we sought to identify dominant dietary patterns and evaluate how well they meet pregnancy nutritional requirements. Nutrient adequacy metrics such as MAR, specific nutrient adequacy metrics, and a dietary TAC score were employed to validate and contextualize the identified dietary patterns. This comprehensive approach aimed to capture both predefined healthy eating guidelines and actual eating behaviors derived from the data, fulfilling the goal of a thorough assessment of diet quality and adequacy in this population.

Inconsistent with global trends, we observed that fewer than half of the women began pregnancy at a normal BMI, where, according to recent findings, this trend was presumed to be lower^22^. Findings from previous domestic studies also demonstrate controversy. In a study by Abdi et al., the prevalence of overweight/obesity in “Bandar Abbas, Iran”, a city in the southern parts of Iran, was about 35%, which is far lower than our results^23^. Nutritionally, excess pre-pregnancy weight and excessive gestational weight gain can both reflect and exacerbate poor dietary quality – diets high in energy-dense, nutrient-poor foods – and increase the risk of complications^24,25^. In line with previous findings, more than half of the population was facing Vitamin D Deficiency (VDD)^26^, which is known to be linked to many pregnancy and offspring conditions^27^. Also, more than 40% of participants had at least 1 of the Metabolic Syndrome (MetS) components, and about 4% of them had more than 3 of the components, crucial to define a MetS case, before the onset of the pregnancy^12,28^.

Daily calorie intake was mainly accumulated in refined grains, dairy products, and fruits (~ 60%), which are staple food choices in Iranian dishes^29,30^. Thereby, a smaller fraction of total energy intake (TEI) in pregnant women is derived from high biological value (HBV) protein sources like red meat, poultry, fish, and eggs (~ 6%). This tendency towards nutrient-poor and calorie-rich food choices has been reported in a study on Middle Eastern pregnant women of the same ages as our participants, where only 12% of the population met the food groups recommended adequate intakes, and the majority (~ 80%) had severely or moderately inadequate food intakes^31^.Overall assessment of the nutritional status of the mothers declares that beneficial minerals and nutrients are not sufficiently consumed through diet, which, to the best of our knowledge, are associated with health complications and neonates’ growth and development^32^. In addition to that, higher sodium intake in comparison to potassium is one of the downsides to this evaluation that aligns with reports from both developing and high-income countries^33^.

As shown in Table 4, pre-defined patterns show a consensus on the inadequate quality of the mothers’ dietary status. NRFs clearly demonstrate that the maternal limiting nutrients exceed the beneficial ones. This is also supported by MDS, where the overall score does not surpass the half (Mean MDS = 3.35). Although the mediterranean diet itself was found to be associated with inadequacies in the number of vital minerals and nutrients^34^, studying it further with NRFs suggests that higher adherence to MD may improve mothers’ total nutrient intake and hence the offspring’s health^35^.

Analysis of food group intakes revealed clear intra-correlations, indicating that the diets of pregnant women clustered into distinct patterns (Fig. 1). For instance, women with high intakes of fruits and vegetables often consumed more dairy products and whole grains, indicating a cohesive “prudent” or health-conscious eating behavior^36^. In contrast, individuals who frequently consumed refined grains, sweetened beverages, and processed snacks tended to have lower intakes of fruits and vegetables, indicating a more “Western” dietary pattern^37^. This signifies that certain foods tend to be eaten together or in place of others and are not random assortments of foods^38^.

The internal validation using a priori and nutritional adequacy metrics presents a tangible shift from best (“Prudent” =PA2) to worst (“Western” =PA3) when the components of the patterns are substituted with less nutritionally cautious desires. Even though none of the statistically significant patterns were thoroughly healthy or unhealthy, the more “Western” components contributed in a diet pattern, the less nutritious it became (Table 6).

From the perspective of nutritional adequacy, TAC, MAR, PAR, and MDS were profoundly consistent with more reliance on the “Prudent” pattern (Fig. 3). In practical terms, women with more Mediterranean-like or nutrient-dense diets were much more likely to meet or exceed the recommended intakes for critical nutrients, especially protein^39^. This is consistent with external evidence: in a cohort of Spanish pregnant women, those with high Mediterranean diet adherence had dramatically lower prevalence of inadequate micronutrient intakes (e.g., only ~ 2.5% had insufficient calcium intake) compared to those with low adherence (~ 27% inadequate)^34^.

Adequate intake of key micronutrients such as iron, folate (vitamin B9), and vitamin B12 is crucial for preventing maternal anemia and supporting the high demands of fetal growth^40^.

A nutrient-dense diet, such as the prudent/Mediterranean pattern, supplies folate from green vegetables and legumes and B12 from lean animal proteins^39^. Diets high in MUFAs (e.g., from olive oil, nuts, and fish) and low in SFAs (from butter, fatty meats, and processed snacks) are known to improve lipid profiles and enhance insulin sensitivity, which in pregnancy assists the protection against gestational diabetes mellitus (GDM) and excessive gestational weight gain^41^. The presumed mechanism is that a diet low in saturated fat and high in fiber and unsaturated fats helps regulate maternal blood sugar levels and prevents excessive maternal weight gain, thereby mitigating one of the major risk factors for GDM^42^. By contrast, diets abundant in fruits, vegetables, and whole grains (as in the healthy pattern) provide more fiber and have a lower glycemic load, supporting satiety and stable blood sugar, thus helping prevent excessive weight gain^43^. Additionally, adequate calcium intake is thought to reduce the risk of hypertensive disorders^44^.

As well as metabolism, organ and systemic function is predominantly supported by a nutritionally qualified diet, like maternal dietary total antioxidant capacity (TAC) in pregnancy, when the body is more prone to increased oxidative stress, and conditions like pre-eclampsia are partly driven by oxidative damage and endothelial dysfunction^45^. The healthy dietary pattern – high in fruits, vegetables, whole grains, and nuts – naturally supplies a broad array of antioxidants and phytochemicals (e.g., vitamin C, flavonoids, carotenoids), whereas the Western pattern, low in those foods, provides fewer antioxidants and more pro-oxidant^46^.

Our findings resonate with, and add to, a growing body of literature on dietary patterns in pregnancy. For instance, a case-control study in Urmia, Iran (northwestern region) extracted three dietary patterns virtually identical to ours – “Healthy,” “Western,” and “Traditional” – and reported that diet quality had a clear relationship with pregnancy outcomes^47^. In that study, higher adherence to the healthy pattern and the local traditional pattern was associated with substantially lower risk of pre-eclampsia, whereas adherence to the Western pattern increased pre-eclampsia odds nearly six-fold^47^.

Another recent hospital-based study in Rasht (northern Iran) found the same three dominant patterns (traditional, Western, healthy) among pregnant women^48^. They observed that women with high Western-pattern scores tended to have greater gestational weight gain and shorter newborn length, reinforcing our emphasis on limiting ultra-processed, high-sugar foods for better pregnancy health^48^. Moreover, meta-analyses of observational studies worldwide indicate that healthy dietary patterns can modestly but significantly lower the risk of gestational diabetes and hypertensive disorders, concluding that adherence to Mediterranean, prudent, or vegetable-rich patterns reduced GDM risk by ~ 14–29%, whereas a Western pattern increased GDM risk by 27%^49^.

This study has several notable strengths. First, it is one of the few to comprehensively assess pregnant women’s diets using both A priori and A posteriori approaches simultaneously. Second, our incorporation of nutrient adequacy metrics as an internal validation tool is a major strength. Third, the study addresses the pregnant population that has been underrepresented in the literature. Despite these strengths, certain limitations must be acknowledged. Most importantly, the study design was cross-sectional, assessing diet and nutritional status at a single mid-pregnancy time point. The lack of neonatal outcome data in our analysis is a related limitation. Our target population was from a particular region in northern Iran; they likely share certain cultural dietary habits, socioeconomic background, and healthcare access that may not reflect pregnant women elsewhere in Iran or in other countries. Thus, there is a need for broader population validation. Additionally, our reliance on an FFQ for dietary assessment introduces the possibility of recall bias and measurement error. In line with this, a prospective study design would strengthen causal inference: for instance, recruiting women in early pregnancy and monitoring how changes in diet patterns affect gestational weight gain trajectory, incidence of GDM, or birth outcomes would be a valuable next step.

## Conclusion

In conclusion, this study provides a thorough look at what pregnant women in northern Iran are eating and how it stacks up nutritionally. Pregnant women enrolled in this study tend to consume less nutritionally and healthily, and it is reflected in their poor mineral and nutritional profile. Adequate protein intake, fiber, and total calories were notable; however overall diet pattern was leaning towards increasing risks in developing major maternal and newborn complications for both before and after pregnancy.

## Supplementary Information

Below is the link to the electronic supplementary material.

Supplementary Material 1

## Data Availability

The datasets used and/or analyzed during the current study are available from the corresponding author on reasonable request.
