# Supplementary material for: Assessment of the dietary and nutritional status of Iranian pregnant women: a study employing both a priori and a posteriori analytical approaches
Source: Sci Rep. 2026 May 21;16:23228. doi: 10.1038/s41598-026-51749-8 (PMC13402745; doi:10.1038/s41598-026-51749-8)
Supplement: Supplementary file 1 — Supplementary Material 1 [file 41598_2026_51749_MOESM1_ESM.docx]

**Supplementary Chart 1.** participant flow chart steps

Pregnant women who visited public prenatal clinics at Mazandaran University of Medical Sciences from March 2020 to February 2023

Eligibility assessment

Met inclusion criteria criteria

Did not meet the criteria

Asked if interested in participating in the study and being involved in assessment interviews

- Being pregnant for more than 14 weeks
- Having medication for chronic diseases
- Being consulted for any sort of diets or special food regimens
- Being diagnosed with any psychological issues

Agreed to enroll

Did not agree to enroll

Completed the dietary and other variable questionnaires

Valid dietary report

dietary energy report was either overestimated or underestimated (800 kcal/day to 5000 kcal/day)

Excluded from food patterns analyses
(N = 10)

Included in non-food pattern analyses
(N = 233)

Included in food patterns analyses
(N = 223)

| **Supplementary Table 1**. A priori diet patterns and nutritional adequacy ratios: definition and details | | |
| --- | --- | --- |
| **Index** | **Definition** | **Reference** |
| MAR | is the average of the nutrient adequacy ratios (NARs) for a set of essential micronutrients. The individual’s NAR is the ratio of intake to the RDA for that nutrient, truncated at 1.0 (100%), so that exceeding the requirement does not inflate the score. MAR thus produces a score between 0 and 1 (or 0–100% when expressed as a percentage), indicating the overall proportion of essential nutrient requirements being met. A higher MAR signifies better micronutrient adequacy of the diet | Steyn (2014), Keli Shadi (2019), Ladipo (2000), Hatløy (1998), Awoke (2022) |
| PAR | We calculated a Protein Adequacy Ratio for each participant. The PAR was defined analogously to a NAR for protein: we took the woman’s average daily protein intake (g/day) and divided it by her estimated protein requirement. The individual requirement was computed as 1.2 grams of protein per kilogram of pre-pregnancy body weight per day during the early (∼16 weeks) stages of pregnancy. We then expressed PAR both as a continuous value and in categories (e.g., <1 vs. ≥1, indicating inadequate vs. adequate protein intake) | Elango (2016), Stephens (2015) |
| TAC | Dietary TAC was calculated using a ferric-reducing antioxidant power (FRAP) assay-based nutrient database: essentially, each food item’s contribution to antioxidant capacity (in mmol FRAP equivalents per 100g, as reported in the literature) was multiplied by the amount of that food in the diet and summed across all foods to yield a total daily antioxidant capacity. Because TAC values are unit-dependent and can vary in scale, we standardized each individual’s TAC to a z-score. The TAC z-score was computed by subtracting the sample mean TAC and dividing by the standard deviation, so that a value of 0 represents the average dietary antioxidant level in the study and +1 or -1 represents one standard deviation above or below the mean, respectively. | Wang (2012), Sadowska-Bartosz (2022), Ha (2023) |
| MUFA/SFA | This ratio serves as an index of dietary fat quality. We computed MUFA/SFA from the grams of monounsaturated and saturated fatty acids in the diet. | Asghari (2017), Asghari (2016) |
| Ca/P | The calcium-to-phosphorus ratio is crucial for maintaining bone health and regulating mineral metabolism. We calculated Ca/P as daily calcium (mg) divided by daily phosphorus (mg) intake. | Laskey (1991) |
| Na/K | A lower sodium-to-potassium ratio (ideally <1) is beneficial for cardiovascular health. We computed Na/K as total sodium (mg) divided by total potassium (mg) in the diet. | Vulin (2022), Bailey (2016) |
| B9/B12 | We examined the ratio of folate to vitamin B12 intake. This ratio, as well as aforementioned metrics, was obtained through dividing daily folate (μg dietary folate equivalents) by daily vitamin B12 (μg). |  |
| MDS | Adherence to a Mediterranean-like diet was quantified on a scale of 0 to 9 points (original MDS) based on nine dietary components. These components included high consumption of vegetables, fruits and nuts, legumes, and cereals (traditionally unrefined); high intake of olive oil (reflected in a high monounsaturated-to-saturated fat ratio); moderately high fish intake; low to moderate intake of dairy (mostly cheese/yogurt); low consumption of red meat and poultry; and moderate alcohol intake (primarily wine with meals). In the present study, we adapted the MDS to the Iranian context by omitting alcohol as a component, since alcohol consumption is negligible for cultural/religious reasons (40, 45). Thus, the modified MDS had eight components with a total score ranging from 0 (minimal adherence) to 8 (maximal adherence). Each participant’s diet was scored 0 or 1 point for each component using conventional cut-offs (e.g. sex-specific median intakes): one point was assigned if the intake of a beneficial food group (vegetables, fruits, legumes, nuts, etc.) was above the median, or if the intake of a detrimental food (e.g. red meat) was below the median, consistent with the original MDS protocol. For the fat quality component, we calculated the ratio of monounsaturated to saturated fatty acids (MUFA/SFA) and awarded 1 point for a ratio above the study median (40). The component points were then summed to yield the total MDS for each individual (higher scores indicate closer adherence to the traditional Mediterranean diet pattern). | Mirmiran (2015), Moustakim (2023) |
| NRFI | We assessed overall diet quality via three versions of the Nutrient-Rich Food index, which is a nutrient profiling method that balances nutrients to encourage against nutrients to limit (Supplementary Table 3.). Following the established algorithms by Drewnowski et al, we computed NRF6.3, NRF9.3, and NRF15.3 scores for each participant using the nutrient intake data from Nutritionist IV. Briefly, the NRF6.3 index included 6 beneficial nutrients – protein, dietary fiber, vitamin A, vitamin C, calcium, and iron – and 3 nutrients to limit – saturated fat, added sugar, and sodium. For each of the six encouraged nutrients, we calculated the percentage of the daily recommended value (%DV) provided by the person’s diet (truncated at 100% for any nutrient exceeding the recommendation), and similarly calculated %DV for the three limiting nutrients. The NRF6.3 score was then obtained by summing the %DV of the encouraged nutrients and subtracting the %DV of the limiting nutrients. The NRF9.3 index was calculated in the same manner but with an expanded list of 9 nutrients to encourage: it included all components of NRF6.3 plus vitamin E, magnesium, and potassium. The NRF15.3 (sometimes called “extended” NRF) further broadened the nutrient profile, covering 15 nutrients to encourage – it encompassed the original six along with additional vitamins and minerals important in the diet (such as vitamins D, E, B_1, B_2, folate (B_9), vitamin B_12, plus magnesium, zinc, potassium, and also accounted for unsaturated fat by including monounsaturated fat) – again balanced against the same 3 limiting nutrients (saturated fat, sugar, sodium). All NRF scores were computed per individual by applying the above formulas to that person’s daily nutrient intakes. This yielded a continuous score for each NRF variant, where higher values reflect a more nutrient-dense, high-quality diet (i.e., higher intakes of protein, fiber, and vitamins/minerals relative to calories, and lower intakes of saturated fat, sugars, and sodium). | Fulgoni (2009), Sluik (2015), Drewnowski (2010) |
| **Abbreviations**: Mean Adequacy Ratio (MAR), Protein Adequacy Ratio (PAR), Dietary TAC (Total Antioxidant Capacity), MUFA/SFA ratio (Monounsaturated Fat to Saturated Fat), Ca/P ratio (Calcium-to-Phosphorus), Na/K ratio (Sodium to Potassium), B9/B12 ratio (Folate to Vitamin B12), Mediterranean Diet Score (MDS), Nutrient-Rich Food Indices (NRF6.3, NRF9.3, NRF15.3) | | |

| **Supplementary Table 2.** List of micronutrients and their respective Dietary Reference Intakes for Nutrient Adequacy Ratio (NAR) and Mean nutrient Adequacy Ratio (MAR) | |
| --- | --- |
| **Nutrients (units)** | **RDA** |
| Sodium (mg) | 1500.00 |
| Potassium (mg) | 4700.00 |
| Iron (mg) | 27.00 |
| Calcium (mg) | 1000.00 |
| Magnesium (mg) | 350.00 |
| Phosphorus (mg) | 700.00 |
| Zinc (mg) | 11.00 |
| Copper (mg) | 1.00 |
| Manganese (mg) | 2.00 |
| Selenium (mcg) | 60.00 |
| Fluoride (mg) | 3.00 |
| Chromium (mcg) | 30.00 |
| Vitamin A (mcg RAE) | 770.00 |
| Beta-carotene (mcg) | 770.00 |
| Vitamin E (mg AT) | 15.00 |
| Alpha-tocopherol (mg AT) | 15.00 |
| Vitamin B1 -Thiamin- (mg) | 1.40 |
| Vitamin B2 -Riboflavin- (mg) | 1.40 |
| Vitamin B3 -Niacin- (mg) | 18.00 |
| Vitamin B6 (mg) | 1.90 |
| Folate (mcg DFE) | 600.00 |
| Vitamin B12 (mcg) | 2.60 |
| Vitamin B5 -Pantothenic Acid- (mg) | 6.00 |
| Biotin (mcg) | 30.00 |
| Vitamin C (mg) | 85.00 |
| Vitamin D (IU) | 600.00 |
| Vitamin K (mcg) | 90.00 |
| fiber (g) | 28.00 |
| Iodine (mcg) | 220.00 |
| **Abbreviations**: Recommended Dietary Allowance = RDA | |

| **Supplementary Table 3.** List of micronutrients and their respective Dietary Reference Intakes for Nutrient Rich Food Indexes | | | | |
| --- | --- | --- | --- | --- |
| **Beneficial Components ^a^** | | | **Limiting Components** | |
| **NRF15.3** | **NRF9.3** | **NRF6.3** | **Nutrient** | **UL** |
| Protein | Protein | Protein | Sodium | 2300 mg/day |
| fiber | fiber | fiber | Saturated fat | 20 g/day |
| Vitamin A | Vitamin A | Vitamin A | Added sugar | 50 g/day |
| Vitamin C | Vitamin C | Vitamin C | Cholesterol | 300 mg/day |
| Calcium | Calcium | Calcium |  | |
| Iron | Iron | Iron |  |  |
| Vitamin E | Vitamin E |  |  |  |
| Magnesium | Magnesium |  |  |  |
| Potassium | Potassium |  |  |  |
| MUFA |  |  |  |  |
| Vitamin D |  |  |  |  |
| Vitamin B1 |  |  |  |  |
| Vitamin B2 |  |  |  |  |
| Vitamin B9 |  |  |  |  |
| Vitamin B12 |  |  |  |  |
| Zinc |  |  |  |  |
| **Abbreviations**: Upper Limit = UL; Nutrient Rich Food = NRF, Monounsaturated Fatty Acids = MUFA  ^a^ We applied the Recommended Dietary Allowance (RDA) values for beneficial components of NRF presented in Supplementary Table 1. | | | | |

| **Supplementary Table 4**. Hyperparameters of the PCA model for drawing A posteriori dietary patterns from 26 food groups consumed by participants | | |
| --- | --- | --- |
| **Hyperparameter** | **Value** | **Explanation** |
| nfactors | 3 | Number of principal components retained |
| rotate | "promax" | Oblique rotation allows correlated components |
| fm | "pa" | Principal Axis Factoring, ideal for non-normal or real-world data |
| cor | TRUE (default) | Uses a **correlation matrix** for factor analysis |
| cut (for display) | 0.3 (threshold, optional) | Threshold for displaying "significant" loadings in visualizations |
| data source | Filtered from 26 food items | Based on observed intake patterns |
| variance captured | ~55% cumulatively (simulated) | Total variance explained by the 3 retained components |
| scores (if generated) | Not generated (optional) | Can be added if subject-level scores are needed |
